# Label Statements and Perceived Health Benefits of Dietary Supplements

**DOI:** 10.1001/jamanetworkopen.2025.33118

**Published:** 2025-09-22

**Authors:** Joanna Nicole Assadourian, Eric D. Peterson, Ann Marie Navar

**Affiliations:** 1 University of Texas Southwestern Medical Center, Dallas; 2 Duke University Medical Center, Durham, North Carolina

## Abstract

**Importance:**

Dietary supplement use is widespread, and their labels often contain structure/function claims (eg, supports heart or brain health), which legally are not intended to imply the supplement prevents or treats a disease. How structure/function statements are interpreted by consumers, however, is unknown.

**Objectives:**

To evaluate how commonly used structure/function claims are associated with consumer beliefs regarding the health benefits of supplements.

**Design, Setting, and Participants:**

We conducted 2 online survey studies of US adults participating in the SurveyMonkey Audience platform, one for a fish oil supplement and one for a hypothetical supplement called Viadin H. For each survey, participants were randomized to 1 of 4 labels that were otherwise identical but had different health-related label statements (eg, supports heart health, cognitive function) and then asked about the supplement’s health benefits.

**Main Outcomes and Measures:**

The percentage of participants who reported that the supplement was somewhat likely or very likely to prevent or treat a list of diseases.

**Results:**

Overall, 2239 participants completed the fish oil survey (473 [21.5%] aged 45-54 years; 1142 [52.6%] women), and 2164 completed the Viadin H survey (510 [23.7%] aged 45-54 years; 1085 [51.2%] women). Respondents shown the fish oil label that said “Supports Heart Health” were more likely than those shown a no-claim control to report that it prevents heart attacks (370 of 592 [62.5%] vs 306 of 568 [53.9%]; *P* = .003) and heart failure (349 [59.0%] vs 288 [50.7%]; *P* = .005). Those shown the “Supports Cognitive Function” label claim were more likely to report that fish oil prevents dementia (255 of 538 [47.4%] vs 225 [39.6%]; *P* = .009) and improves memory in dementia (258 [48.0%] vs 230 [40.5%]; *P* = .01) compared with those shown no-claim labels. For Viadin H, those shown a label stating “Heart Health” or “Supports Heart Function” were more likely to report the supplement prevents heart attack or heart failure, while those shown a label stating “Brain Health” or “Supports Cognitive Function” were more likely to report the supplement prevented dementia or improved memory (eg, lower risk of heart attack: “Heart Health” label, 209 of 525 [40.0%]; “Supports Heart Function” label, 225 of 556 [40.5%]; “Brain Health” label, 113 of 556 [20.2%]; and “Supports Cognitive Function” label, 124 of 536 [23.3%]; *P* < .001).

**Conclusions and Relevance:**

In this survey study, commonly used structure/function claims on supplement labels were often misinterpreted by consumers to imply a disease-specific benefit, contrary to their regulatory intent. Improved regulations for supplement labeling may be needed to minimize consumer misunderstanding.

## Key Points

**Question:**

Do statements on nutritional supplement labels affect how consumers perceive the supplement’s health benefits?

**Findings:**

In this survey study, 4403 US adults were randomized to see different labels for fish oil and a nonexistent, hypothetical supplement. For both supplements, participants who viewed labels with statements such as “heart health” or “brain health” were more likely to report believing that the supplement prevented or treated cardiac- and neurologic-related conditions.

**Meaning:**

In this study, consumers were more likely to report specific disease-related health benefits from supplements when their labels included commonly used statements that exceed the scope of their intended meaning, supporting the need to reevaluate regulations regarding nutritional supplement labeling.

## Teaser

This survey study evaluates how consumers interpret statements such as “supports heart health,” which often appear on the labels of dietary supplements.

## Introduction

Supplement use in the United States is increasingly common, with 56% of US adults reporting that they take 1 or more dietary supplements daily.^1^ Many supplement users take these under the belief that the supplement prevents or treats specific diseases or health conditions.^2,3,4^ One possible driver of these consumer beliefs may be statements made on the supplement labels.

Three types of label claims are allowed under the US Food and Drug Administration (FDA) regulation: nutrient claims, health claims, and structure/function claims.^5^ Nutrient claims refer to the quantity of an ingredient in the dietary supplement, eg, “only 200 mg of sodium.” Health claims refer to any statement that associates a supplement ingredient to the treatment or prevention of a specific disease based on evidence review by the FDA; these are relatively rare. Structure/function claims are intended to refer to the role of an ingredient in the supplement affecting normal function of the human body, such as “calcium builds strong bones.” These claims are not intended to describe that taking the supplement itself will prevent or treat a disease. Nevertheless, the language used in structure/function claims is often ambiguous, such as “heart health” or “supports cognitive function.”^6^ The Office of Inspector General and the Government Accountability Office have raised the possibility that these vague structure/function claims could lead to consumer misinformation.^7,8^ However, the report’s recommendations to strengthen the FDA’s authority in regulating structure/function claims have not been implemented in part due to lack of empirical evidence.

Multiple large randomized clinical trials have found that fish oil supplements do not prevent cardiovascular events and are not recommended for primary or secondary prevention of atherosclerotic cardiovascular disease (ASCVD).^9,10,11,12^ Despite this, nearly 20% of adults in the United States with ASCVD take a fish oil supplement, the majority of whom do so for heart health.^4^ Nearly three-quarters of fish oil supplements on the market have at least 1 health-related structure/function claim.^6^ Whether and how these statements may influence consumer perceptions regarding the supplement’s impact on prevention or treatment of disease remains unclear.

Therefore, we sought to evaluate the degree to which commonly used structure/function claim language related to heart or brain health is associated with potential consumer perceptions regarding supplements using 2 different randomized surveys. In each survey, participants were randomized to see supplement labels that varied only by the presence or absence of a structure/function statement. The first survey used fish oil, as it is among the most commonly used supplements in the United States. In the second, to ensure participants had no prior or outside knowledge about the supplement, we created a hypothetical supplement that is not currently on the market.

## Methods

The reporting of this survey study followed the American Association for Public Opinion Research (AAPOR) reporting guideline. The study team developed the 2 randomized surveys, which were deployed to the SurveyMonkey Audience platform, whose members include individuals who have agreed to be contacted for survey research. Email invitations, in English, were sent from the platform, and individuals opted in to participate. The email sent to participants was a general template used for all surveys and stated, “We’re conducting a survey and your input would be appreciated. Click the button below to start the survey. Thank you for your participation!” with the subject line “We Want Your Opinion.” The platform panelists may opt to take surveys for charity, the opportunity for sweepstakes prizes, or credits that are redeemable for gift cards or charity donations. Our study team did not provide an additional incentive for participation. This panel was selected because it mirrors that used by the FDA in a previous study evaluating interpretation of health claim language.^13^

Eligible participants were aged 18 years or older living in the United States who had opted to participate in the platform. Participants were randomly selected by age and sex according to the US census by the platform to attempt to generate a sample that is similar in age and sex to the US population. No additional weighting was used. Neither survey collected any participant identifiable information. Per the Common Rule, informed consent was not required for this anonymous survey. An introduction page to the survey informed participants, “Your response is voluntary and will remain anonymous to the survey collectors.” The platform does not track the number of participants emailed and invited to participate in the survey, only the number who agree to participate. Full text of the survey questions used, which was available only in English, is available in the eAppendix in Supplement 1.

Both surveys were designed to collect at least 1552 responses across the 4 label groups (388 participants in each group) to provide 80% power to detect at least a 10% absolute difference in the rate of perceived health benefits between groups, using a 2-sided *Z* test for proportions with an alpha of .05 and power of 0.8. We further increased our sample size estimates by 20% to account for potential nonresponse for a target of at least 1862. The survey was programmed such that participants in the fish oil survey were not eligible to participate in the Viadin H survey. The fish oil survey was performed from January 26 to 27, 2024, and the Viadin H survey was performed on March 27 to 28, 2024. This anonymous study was deemed exempt from institutional review board review by the UT Southwestern institutional review board.

### Survey 1: Fish Oil

We created a label for a hypothetical fish oil supplement called “Nature’s Fish Omega-3 Fish Oil.” This supplement was chosen given its pervasive use and numerous negative randomized clinical trials indicating no cardiovascular benefit, with few exceptions. The label was designed to emulate current on-market fish oil labels. Four different versions of the same label were created that varied only in whether and what type of health claim was made. The first 2 contained different structure/function claims: (1) supports heart health (eFigure 1 in Supplement 1) and (2) supports cognitive function (eFigure 2 in Supplement 1) based on prior research on types of structure/function claim for cardiovascular and neurologic systems on fish oil supplements.^6^ The third label contained actual FDA-approved qualified health claim (QHC) language for fish oil’s possible positive association with coronary heart disease: (3) “may reduce risk of coronary heart disease” (eFigure 3 in Supplement 1) on the front. This label also included the FDA-mandated language qualifying the health claim in smaller font on the back: “supportive, but not conclusive research shows that consumption of EPA and DHA omega-3 fatty acids may reduce the risk of coronary heart disease.” The fourth label was a control label that had no health claim (eFigure 4 in Supplement 1).

Participants were randomized to see only 1 of the 4 different labels to review and then answer questions based on the label information presented about the potential health benefits of the supplement. Participants were asked, “On a scale of 1 to 5, how likely is it that the product may help lower or reduce the risk of any of these health problems” for the following disease states: heart attack, stroke, dementia, heart failure, hyperlipidemia, osteoporosis/low bone density, or cancer. Osteoporosis and cancer were included as negative controls given absence of association or known benefit for prevention or treatment by fish oil. These questions were modeled on a previous supplement survey study conducted by the FDA.^13^ The order of the disease states was randomly reshuffled between respondents. A 5-point Likert scale was used for respondents to capture the degree of perceived likelihood from 1 (not at all likely) to 5 (very likely). All questions included an option of “Don’t know/prefer not to answer.” Among responders, the proportions of responding likely or very likely were calculated for each group and compared with those in the control arm.

### Survey 2: Viadin H

To avoid any anchoring on prior knowledge, the second survey evaluated different label statements for a hypothetical supplement called Viadin H that the respondents could not have any preexisting beliefs about. Parallel to the prior fish oil study design, 4 versions of the Viadin H label were created, each with a different structure/function claim: (1) “Heart Health,” (2) “Supports Heart Function,” (3) “Brain Health,” and (4) “Supports Cognitive Function” (eFigures 5-8 in Supplement 1). Participants were randomly shown 1 of the 4 labels and asked about the likelihood that Viadin H would prevent or treat the same list of diseases as in survey 1.

We hypothesized that participants shown the heart-related labels would be more likely to report a cardiovascular benefit of Viadin H, while those shown labels related to brain health or cognitive function would be more likely to report that Viadin H prevents stroke or dementia. Cancer and osteoporosis were included as negative control conditions, and the order of conditions was randomly shuffled between respondents.

For both surveys, participants reported their own supplement use. There were optional demographic questions regarding age, self-reported race and ethnicity, gender, education level, household income, and health conditions.

### Statistical Analysis

Descriptive analyses were used to describe characteristics of the overall participant responses to questions regarding knowledge of each supplement and perceived health benefits. We used χ^2^ tests to compare differences in proportions between groups with a 2-sided threshold of *P* < .05 for statistical significance. For the fish oil survey, responses were compared between each label group and the control label. For the Viadin H survey, because no control group was created, differences in health perceptions between groups shown the heart-related labels and those shown a brain and cognitive function labels were evaluated using χ^2^ tests. Data processing was performed in Excel version 2507 (Microsoft Corp), and data analysis was performed in Stata SE version 18.0 (Stata Corp).

## Results

### Study Populations

Overall, 2275 individuals agreed to participate in the fish oil survey, of whom 2239 (98.4%) completed the survey. A total of 2180 agreed to take the Viadin H survey, of whom 2164 (99.2%) completed it. The Table shows the characteristics of the survey populations. In both the fish oil and Viadin H surveys, there were slightly more women than men (1142 [52.6%] and 1085 [51.2%], respectively) and the largest group of respondents were aged 45 to 54 years (473 [21.5%] and 510 [23.7%], respectively). In the fish oil survey, 234 respondents (10.7%) were Asian, 303 (14.3%) were Hispanic, and 1584 (72.7%) were White. In the Viadin H survey, 209 respondents (9.7%) were Asian, 320 (15.6%) were Hispanic, and 1550 (72.2%) were White. More than half in each group (fish oil, 1238 [57.0%]; Viadin H, 1218 [57.4%]) reported a college degree or higher. Supplement use in both survey populations was also high; 1932 respondents (84.9%) in the fish oil survey and 1687 (79.8%) in the Viadin H survey reported some form of supplement use, with multivitamins and vitamin D being the most common.

**Table.** Characteristics of 2 Survey Populations

Characteristic	Participants by survey, No. (%)	
	Fish oil (n = 2239)	Viadin H (n = 2164)
Age, y		
18-24	204 (9.3)	192 (8.9)
25-34	420 (19.1)	321 (14.9)
35-44	407 (18.5)	450 (20.9)
45-54	473 (21.5)	510 (23.7)
55-64	303 (13.8)	296 (13.8)
65-74	278 (12.6)	269 (12.5)
≥75	116 (5.3)	113 (5.3)
Missing or skipped	74 (3.3)	29 (1.3)
Gender identity		
Woman	1142 (52.6)	1085 (51.2)
Man	955 (44.0)	971 (45.8)
Transgender woman	12 (0.6)	14 (0.7)
Transgender man	10 (0.5)	8 (0.4)
Nonbinary	12 (0.6)	10 (0.5)
Other^a^	6 (0.3)	4 (0.2)
Prefer not to answer	34 (1.6)	29 (1.4)
Missing or skipped	104 (4.6)	59 (2.7)
Race		
American Indian or Alaskan Native	32 (1.5)	40 (1.9)
Asian	234 (10.7)	209 (9.7)
Black or African American	136 (6.2)	165 (7.7)
Multiracial	50 (2.3)	39 (1.8)
Native Hawaiian or Pacific Islander	15 (0.7)	15 (0.7)
White	1584 (72.7)	1550 (72.2)
Other^a^	51 (2.3)	64 (3.0)
Prefer not to answer	77 (3.5)	66 (3.1)
Missing	96 (4.2)	32 (1.5)
Ethnicity		
Hispanic or Latinx	303 (14.3)	320 (15.6)
Education		
Some high school	57 (2.6)	65 (3.1)
High school or GED	312 (14.4)	302 (14.2)
Some college	528 (24.3)	507 (23.9)
College graduate	632 (29.1)	605 (28.5)
Graduate degree	606 (27.9)	613 (28.9)
Prefer not to answer or missing	140 (6.2)	88 (4.1)
Annual income, $		
<20 000	236 (10.9)	211 (9.9)
20 000-39 999	331 (15.3)	286 (13.5)
40 000-59 999	337 (15.6)	311 (14.6)
60 000-79 999	274 (12.7)	300 (14.1)
80 000-99 999	204 (9.4)	221 (10.4)
100 000-149 999	355 (16.4)	383 (18.0)
≥150 000	259 (12.0)	255 (12.0)
Prefer not to answer or missing	279 (12.6)	213 (9.9)
Supplement use		
Any supplement use	1932 (84.9)	1687 (79.8)
Vitamin D	1216 (53.5)	1081 (49.6)
Multivitamin	1174 (51.6)	1115 (51.2)
Fish oil	941 (42.1)	545 (25.0)
Calcium	565 (24.8)	456 (20.9)
Magnesium	529 (23.3)	434 (19.9)
Vitamin B12	517 (22.7)	451 (20.7)
Iron	343 (15.1)	299 (13.7)
B complex	293 (12.9)	256 (11.7)
CoQ10	181 (8.0)	141 (6.5)
Other	15 (7.9)	96 (4.4)

Abbreviations: CoQ10, coenzyme 10; GED, General Educational Development.

^a^ Other was an option provided to participants with no further description.

eTables 1 and 2 in Supplement 1 show characteristics of the fish oil and Viadin H surveys stratified by randomization group. No significant differences were seen in demographic characteristics, education level, income, or supplement use between randomized groups for either survey.

### Fish Oil Survey Results

eTable 3 in Supplement 1 shows participant responses regarding familiarity with and perceptions about fish oil prior to being shown the fish oil label. Most participants reported some familiarity with fish oil, with 956 (42.0%) reporting being somewhat familiar and 867 (38.1%) very familiar with fish oil. Among the 1823 respondents who were somewhat or very familiar with fish oil, 1456 (78.8%) reported that they were aware of a health benefit. Fish oil users had much higher rates of reporting awareness of health benefits of fish oil (747 of 941 [93.7%] vs 696 of 1296 [68.2%]; *P* < .001) than nonusers even before seeing the label (eTable 3 in the Supplement).

**Figure 1.**
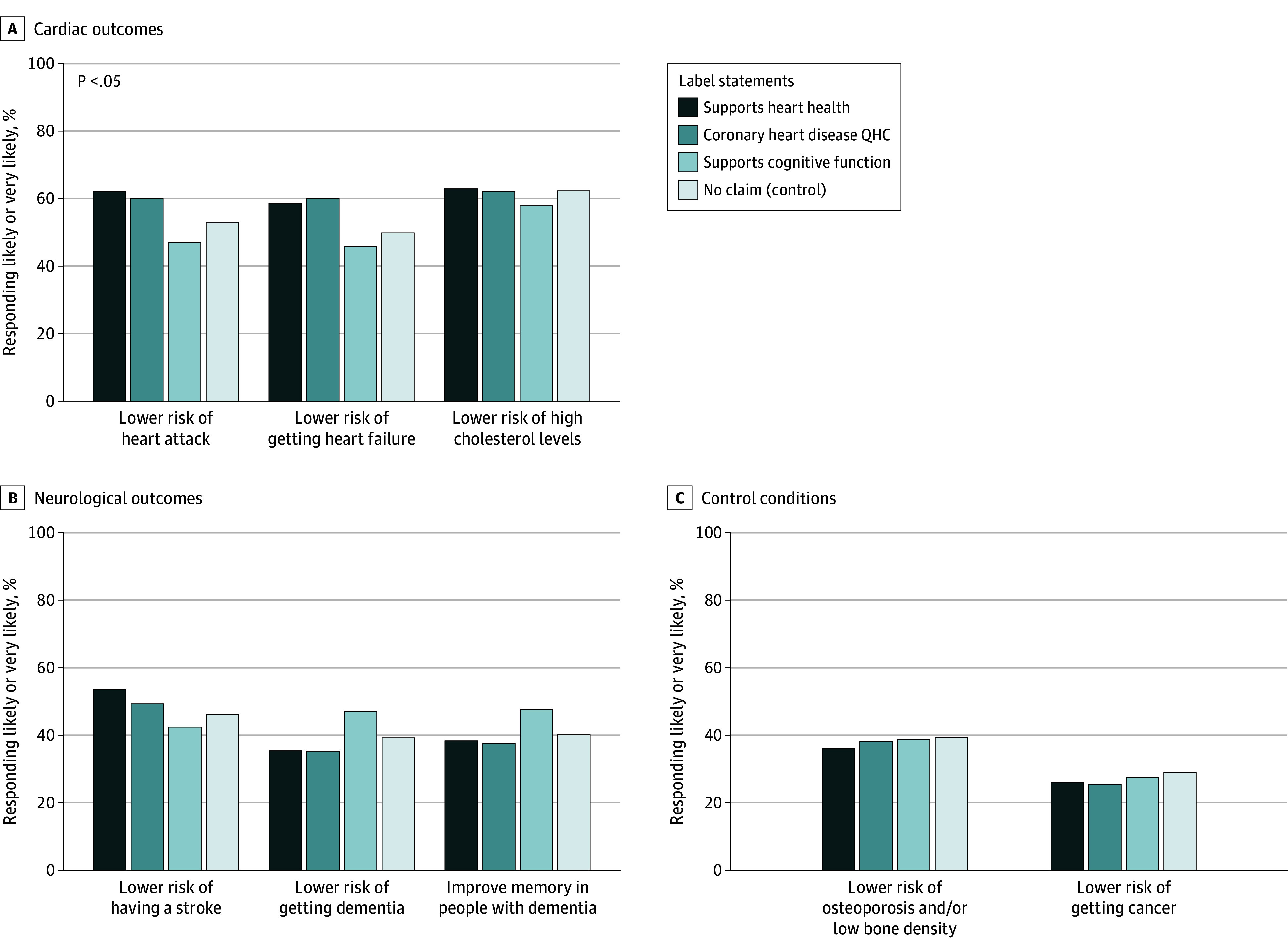
Respondents Who Reported That the Fish Oil Supplement Would Treat or Prevent Specific Diseases, by Randomized Label Statement Group The figure shows the proportion of respondents randomized to each label group that reported that the supplement would likely or very likely lead to the health outcome shown. *P* values represent the difference between each group and the control group shown no label based on χ^2^ tests. QHC indicates qualified health claim.

Figure 1 and eTable 4 in Supplement 1 show the proportion of respondents who reported that fish oil would likely or very likely reduce the risk of specific diseases by randomized label group. Compared with the control group (no health claim), participants shown the “Support Heart Health” label and the coronary heart disease QHC label were more likely to report fish oil would likely lower the risk of a heart attack (support heart health vs control, 370 of 592 [62.5%] vs 306 of 568 [53.9%]; *P* = .003; QHC vs control, 326 of 541 [60.3%] vs 306 [53.9%]; *P* = .03) or developing heart failure (support heart health vs control, 349 [59.0%] vs 288 [50.7%]; *P* = .005; QHC vs control, 326 [60.3%] vs 288 [50.7%]; *P* = .001) but not that fish oil would lower high cholesterol (Figure 1A). Participants shown the “Supports Cognitive Function” label, compared with the control group, were more likely to report that fish oil would lower the risk of dementia (255 of 538 [47.4%] vs 225 [39.6%]; *P* = .009) or improve memory in people with dementia (258 [48.0%] vs 230 [40.5%]; *P* = .01) (Figure 1B). No difference was seen between label groups in the proportion reporting that fish oil would prevent osteoporosis or cancer (Figure 1C). Numerically, more patients reported that fish oil would reduce the risk of heart attack when shown a label with the statement “Supports Heart Health” than the label with the FDA-approved QHC language for coronary heart disease (370 [62.5%] vs 326 [60.3%]).

When stratified by fish oil use, among individuals who regularly used fish oil supplements, no statistically significant differences were seen in reported health benefits by claim type on the label (eTable 5 in Supplement 1). In contrast, among those not taking fish oil, those shown the “Supports Heart Health” label or coronary heart disease QHC label, compared with the control group, were more likely to report fish oil would prevent a heart attack (supports heart health vs control, 187 of 334 [56.0%] vs 145 of 335 [43.3%]; *P* = .001; QHC vs control, 155 of 303 [51.2%] vs 145 [43.3%]; *P* = .047) or heart failure (supports heart health vs control, 181 [54.2%] vs 130 [38.8%]; *P* < .001; QHC vs control, 152 [50.2%] vs 130 [38.8%]; *P* = .004). Similarly, those shown the “Supports Cognitive Function” label, compared with the control group, were more likely to report that fish oil would prevent dementia (116 of 324 [35.8%] vs 86 [25.7%]; *P* = .005) and improve memory in people with dementia (124 [38.3%] vs 94 [28.1%]; *P* = .005).

### Viadin H Survey

**Figure 2.**
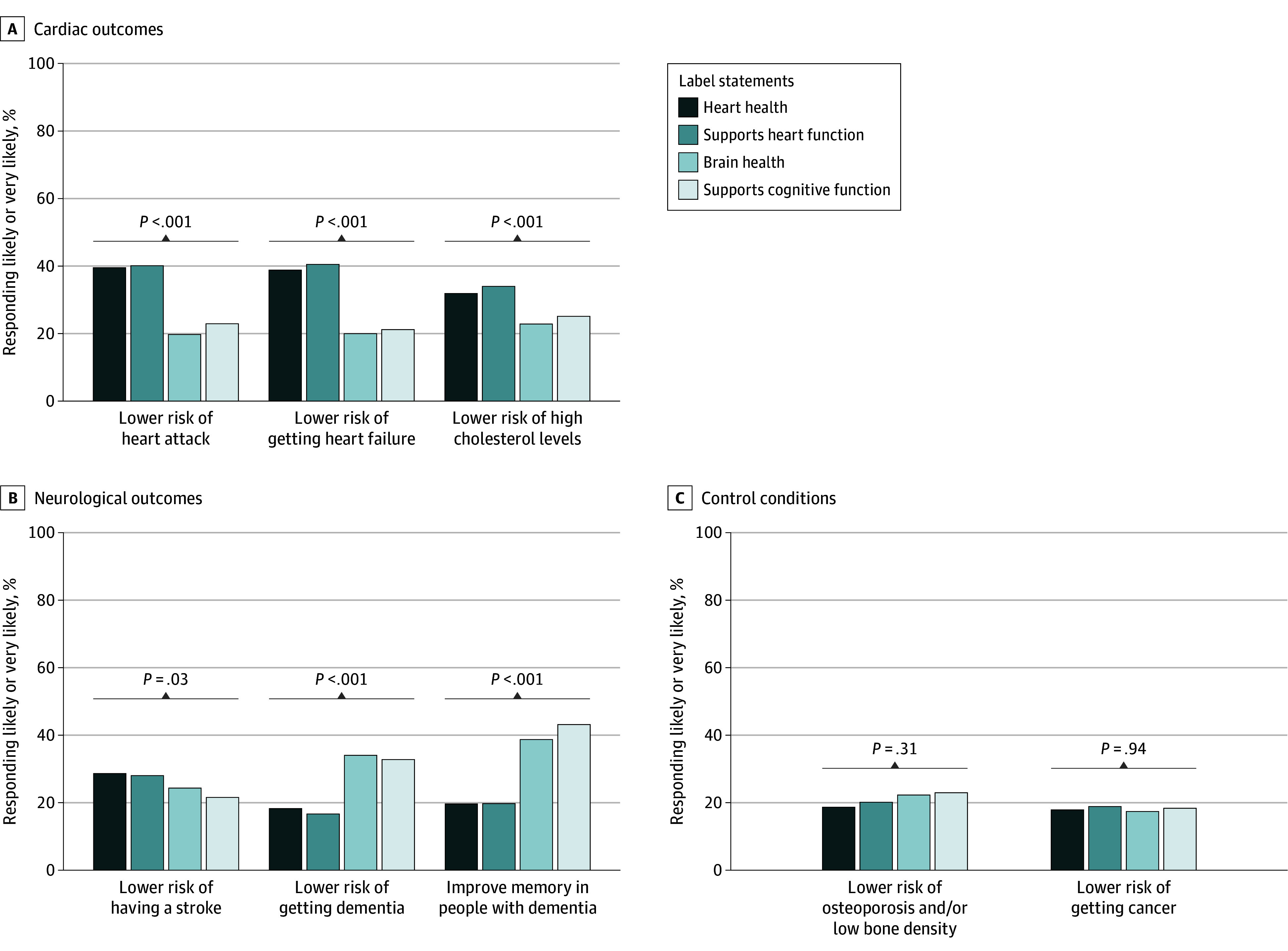
Respondents Who Reported That the Viadin H Supplement Would Treat or Prevent Specific Diseases, by Randomized Label Statement Group The figure shows the proportion of respondents randomized to each label group that reported that the supplement would likely or very likely lead to the health outcome shown. *P* values represent between-group difference based on χ^2^ tests.

Figure 2 and eTable 6 in Supplement 1 present the proportion of individuals in each randomized group who responded that Viadin H would prevent or treat specific health conditions. Statistically significant differences were seen in the proportion of participants reporting benefit for cardiovascular disease (eg, lower risk of heart attack: “Heart Health” label, 209 of 525 [40.0%]; “Supports Heart Function” label, 225 of 556 [40.5%]; “Brain Health” label, 113 of 556 [20.2%]; and “Supports Cognitive Function” label, 124 of 536 [23.3%]; *P* < .001) (Figure 2A) and neurologic diseases (eg, lower risk of getting dementia: “Heart Health” label, 98 [18.7%]; “Supports Heart Function” label, 95 [17.1%]; “Brain Health” label, 193 [34.5%]; and “Supports Cognitive Function” label, 177 [33.3%]; *P* < .001) (Figure 2B) by respective label category, but not for the control conditions of osteoporosis and cancer (Figure 2C).

Responses were similar for those shown the “Heart Health” and “Supports Heart Function” labels and for those shown the “Brain Health” and “Supports Cognitive Function” labels. eFigure 9 in Supplement 1 shows results combining responses for those shown the 2 heart-related labels and those shown the 2 brain-related labels. Overall, among 1081 respondents shown heart related labels, 434 (40.5%) and 432 (40.9%) indicated the supplement would reduce the likelihood of a heart attack or getting heart failure, nearly twice that in those shown the “Brain Health” or “Supports Cognitive Function” label group (heart attack, 237 of 1092 [20.2%]; *P* < .001; heart failure, 229 [20.4%]; *P* < .001,respectively). Similarly, of 1092 participants shown either the “Brain Health” or “Supports Cognitive Function” label, 451 (41.3%) reported the supplement would improve memory in people with dementia, more than twice the number who were shown 1 of the 2 heart-related labels (217 [20.1%]; *P* < .001).

## Discussion

By law, structure/function claims on supplement labels should describe the role of a nutrient in the body and should not state that the supplement prevents or treats any disease. In this survey study, we found that multiple commonly used label statements technically classified as structure/function claims on supplement labels, including heart health, brain health, and cognitive function, were associated with perceptions regarding specific disease benefits. Participants shown supplement labels with statements about heart health and heart function were more likely to report that the supplement could help prevent a heart attack or heart failure, while those shown labels with statements about cognitive support or brain function were more likely to report the supplement prevented dementia or improved cognition in people with dementia. These findings were consistent when participants were shown a label for a widely known supplement, fish oil, and when participants were shown a hypothetical supplement with which respondents had no prior familiarity. Nearly twice the number of respondents reported that a hypothetical supplement called Viadin H would prevent a heart attack when shown a label with the statement “Heart Health” or “Supports Heart Function” than without.

For the fish oil survey, the association of label claims with consumer perceptions did vary by the respondent’s degree of familiarity with fish oil. Those not taking fish oil had greater differences in reported health benefits between randomized label groups than those taking fish oil. Fish oil users likely had greater preestablished beliefs regarding the benefits of fish oil and displayed fewer differences between groups based on the label shown. Notably, when we assessed health beliefs between label statement groups for a purely hypothetical supplement that respondents had no prior knowledge of, differences were more pronounced. We identified a nearly 2-fold difference in reported rates of certain diseases prevented depending on the type of claim language used. For example, placing the words “Heart Health” on the label of a new supplement led a 20–percentage point increase in the proportion of respondents to report it would prevent a heart attack. This suggests that regulation on labels may be most important for new supplements for which consumers have the least familiarity.

While prior research has shown consumers often report taking a supplement because of health reasons, what is actually influencing that belief is less well established and likely multifactorial.^2,3,4^ Our study focused only on one information channel, which was the supplement label itself. Although we did find that survey respondents were more likely to report a disease-related benefit of the supplement when shown just the label, whether this would be sufficient to change behavior regarding taking the supplement is unknown.

Importantly, however, the regulation regarding label statements does not center around whether the statements actually change behaviors regarding the supplement, but how those statements are interpreted. By law, structure/function label statements are not intended to state that the supplement treats or prevents a disease. Given our findings that many individuals who see structure/function statements like heart health, brain health, or cognitive function are inferring disease-specific benefits, these statements should not be permitted under the structure/function category.

### Strengths and Limitations

This study has strengths and limitations. The strengths of this study include a large sample size and randomized design. Furthermore, although our survey was not nationally representative, we utilized the same population panel that was previously utilized by the FDA to guide language for QHC development.^13^ Although we attempted to generate a sample of US adults similar in age and sex to the US population, the resulting population was not fully nationally representative. Notably, the participating population did have higher levels of education than the national average, with more than 55% of respondents reporting that they had a college degree or higher compared with 32% observed in the general population.^14^ Furthermore, approximately 80% of respondents reported using at least 1 dietary supplement, higher than the 56% reported in the general US adult population.^1^ Another limitation is that while the proportion of individuals who reported a benefit for the control conditions (osteoporosis and cancer) was similar across labels, the perceived benefit reported for those was nonzero across all groups. While some may have believed that fish oil prevented cancer or osteoporosis, none should have reported this for Viadin H, a hypothetical supplement we invented for the study and that no one would have heard of before. This suggests that a subset of those who took the survey may have been indiscriminately selecting their answers. We also found higher rates of self-reported fish oil use in those given the fish oil survey compared with the Viadin H survey. Participants did not know the topic of the survey when they agreed to participate, so this is unlikely due to selection bias. Rather, this finding may indicate recall bias in self-reported supplement use. Finally, we cannot determine whether respondents were reporting their true beliefs regarding the supplement or whether they were responding with what they believed may be the right answer.

## Conclusions

In this survey study, structure/function statements on dietary supplement labels were associated with survey participants’ beliefs about the supplement’s health outcomes. Language such as supports heart health, cognitive function, or brain health, commonly used on nutritional supplements, was likely interpreted to imply that the supplement prevents or treats specific diseases, including heart attack, heart failure, and dementia. Given that structure/function claims are by law designed to describe the role of a supplement in the body and not imply treatment or prevention of disease, this type of statement is likely inappropriately classified. Statements regarding the health or function of organ systems may be more appropriately classified as health claims and should undergo the same degree of evidence review as qualified or authorized health claims.

## Notes

Supplement 1. **eFigure 1.** Fish Oil “Supports Heart Health” Label **eFigure 2. **Fish Oil “Supports Cognitive Function” Label **eFigure 3.** Fish Oil Coronary Heart Disease Qualified Health Claim Label **eFigure 4.** Fish Oil No Health Claim Label **eFigure 5.** Viadin H Heart Health Label **eFigure 6.** Viadin H Supports Heart Function Label **eFigure 7.** Viadin H Brain Health Label **eFigure 8.** Viadin H Supports Cognitive Function Label **eFigure 9.** Percentage of Respondents That Responded That the Viadin H Supplement Would Treat or Prevent Specific Diseases by Randomized Label Statement Group **eTable 1.** Demographic Characteristics Across Different Fish Oil Label Claim Types **eTable 2.** Demographic Characteristics Across Different Viadin H Label Claim Types **eTable 3.** Baseline Perceived Health Benefits of Fish Oil Among Fish Oil Users and Non–Fish Oil Users **eTable 4.** Perceived Health Benefits Across Fish Oil Label Types **eTable 5.** Perceived Health Benefits Between Fish Oil Users vs Nonusers Across Fish Oil Label Types **eTable 6.** Perceived Health Benefits Across Viadin H Supplement Label Types **eAppendix.** Surveys

Supplement 2.
Data Sharing Statement

## References

[1] Mishra S, Stierman B, Gahche JJ, Potischman N. Dietary supplement use among adults: United States, 2017–2018. NCHS Data Brief No. 399; February 2021. Accessed August 12, 2025. https://www.cdc.gov/nchs/products/databriefs/db399.htm 33663653

[2] Kantor ED, Rehm CD, Du M, White E, Giovannucci EL. Trends in dietary supplement use among US adults from 1999-2012. JAMA. 2016;316(14):1464-1474. doi:10.1001/jama.2016.14403 27727382 PMC5540241

[3] Bailey RL, Gahche JJ, Miller PE, Thomas PR, Dwyer JT. Why US adults use dietary supplements. JAMA Intern Med. 2013;173(5):355-361. doi:10.1001/jamainternmed.2013.2299 23381623

[4] Assadourian JN, Peterson ED, Gupta A, Navar AM. Use of dietary supplements among people with atherosclerotic cardiovascular disease in the United States: a population-based analysis from NHANES. J Am Heart Assoc. 2024;13(9):e033748. doi:10.1161/JAHA.123.033748 38700042 PMC11179876

[5] Shalala DE, Henney JE. Regulations on statements made for dietary supplements concerning the effect of the product on the structure or function of the body. 21 CFR §101. Fed Regist. 2000;65(4):1000-1050. Accessed August 12, 2025. https://www.federalregister.gov/documents/2000/01/06/00-53/regulations-on-statements-made-for-dietary-supplements-concerning-the-effect-of-the-product-on-the 11010621

[6] Assadourian JN, Peterson ED, McDonald SA, Gupta A, Navar AM. Health claims and doses of fish oil supplements in the US. JAMA Cardiol. 2023;8(10):984-988. doi:10.1001/jamacardio.2023.2424 37610733 PMC10448371

[7] Office of Inspector General. Dietary supplements: structure/function claims fail to meet federal requirements. October 2, 2012. Accessed August 12, 2025. https://oig.hhs.gov/reports/all/2012/dietary-supplements-structurefunction-claims-fail-to-meet-federal-requirements/

[8] Government Accountability Office. Food labeling: FDA needs to reassess its approach to protecting consumers from false or misleading claims. January 14, 2011. Accessed August 12, 2025. https://www.gao.gov/products/gao-11-102

[9] Aung T, Halsey J, Kromhout D, ; Omega-3 Treatment Trialists’ Collaboration. Associations of omega-3 fatty acid supplement use with cardiovascular disease risks: meta-analysis of 10 trials involving 77 917 individuals. JAMA Cardiol. 2018;3(3):225-234. doi:10.1001/jamacardio.2017.5205 29387889 PMC5885893

[10] Bowman L, Mafham M, Wallendszus K, ; ASCEND Study Collaborative Group. Effects of n-3 fatty acid supplements in diabetes mellitus. N Engl J Med. 2018;379(16):1540-1550. doi:10.1056/NEJMoa1804989 30146932

[11] Manson JE, Cook NR, Lee IM, ; VITAL Research Group. Marine n-3 fatty acids and prevention of cardiovascular disease and cancer. N Engl J Med. 2019;380(1):23-32. doi:10.1056/NEJMoa1811403 30415637 PMC6392053

[12] Nicholls SJ, Lincoff AM, Garcia M, . Effect of high-dose omega-3 fatty acids vs corn oil on major adverse cardiovascular events in patients at high cardiovascular risk: the STRENGTH randomized clinical trial. JAMA. 2020;324(22):2268-2280. doi:10.1001/jama.2020.22258 33190147 PMC7667577

[13] US Food and Drug Administration. Experimental study of qualified health claims: consumer inferences about monounsaturated fatty acids from olive oil, EPA and DHA omega-3 fatty acids, and green tea. March 28, 2024. Accessed August 12, 2025. https://www.fda.gov/food/nutrition-food-labeling-and-critical-foods/experimental-study-qualified-health-claims-consumer-inferences-about-monounsaturated-fatty-acids-1

[14] McElrath K, Martin M. Bachelor’s degree attainment in the United States: 2005 to 2019. US Census Bureau. February 9, 2021. Accessed August 12, 2025. https://www.census.gov/library/publications/2021/acs/acsbr-009.html

