# Supplementary material for: Label Statements and Perceived Health Benefits of Dietary Supplements
Source: JAMA Netw Open. 2025 Sep 22;8(9):e2533118. doi: 10.1001/jamanetworkopen.2025.33118 (PMC12455376; doi:10.1001/jamanetworkopen.2025.33118)
Supplement: Supplement 1. — eFigure 1. Fish Oil “Supports Heart Health” Label eFigure 2. Fish Oil “Supports Cognitive Function” Label eFigure 3. Fish Oil Coronary Heart Disease Qualified Health Claim Label eFigure 4. Fish Oil No Health Claim Label eFigure 5. Viadin H Heart Health Label eFigure 6. Viadin H Supports Heart Function Label eFigure 7. Viadin H Brain Health Label eFigure 8. Viadin H Supports Cognitive Function Label eFigure 9. Percentage of Respondents That Responded That the Viadin H Supplement Would Treat or Prevent Specific Diseases by Randomized Label Statement Group eTable 1. Demographic Characteristics Across Different Fish Oil Label Claim Types eTable 2. Demographic Characteristics Across Different Viadin H Label Claim Types eTable 3. Baseline Perceived Health Benefits of Fish Oil Among Fish Oil Users and Non–Fish Oil Users eTable 4. Perceived Health Benefits Across Fish Oil Label Types eTable 5. Perceived Health Benefits Between Fish Oil Users vs Nonusers Across Fish Oil Label Types eTable 6. Perceived Health Benefits Across Viadin H Supplement Label Types eAppendix. Surveys [file jamanetwopen-e2533118-s001.pdf]

Supplemental Online Content

Assadourian JN, Peterson ED, Navar AM. Label statements and perceived health benefits of dietary supplements. *JAMA Netw Open.* 2025;8(9):e2533118. doi:10.1001/jamanetworkopen.2025.33118

eFigure 1. Fish Oil “Supports Heart Health” Label

eFigure 2. Fish Oil “Supports Cognitive Function” Label

eFigure 3. Fish Oil Coronary Heart Disease Qualified Health Claim Label

eFigure 4. Fish Oil No Health Claim Label

eFigure 5. Viadin H Heart Health Label

eFigure 6. Viadin H Supports Heart Function Label

eFigure 7. Viadin H Brain Health Label

eFigure 8. Viadin H Supports Cognitive Function Label

eFigure 9. Percentage of Respondents That Responded That the Viadin H Supplement Would Treat or Prevent Specific Diseases by Randomized Label Statement Group

eTable 1. Demographic Characteristics Across Different Fish Oil Label Claim Types

eTable 2. Demographic Characteristics Across Different Viadin H Label Claim Types

eTable 3. Baseline Perceived Health Benefits of Fish Oil Among Fish Oil Users and Non–Fish Oil Users

eTable 4. Perceived Health Benefits Across Fish Oil Label Types

eTable 5. Perceived Health Benefits Between Fish Oil Users vs Nonusers Across Fish Oil Label Types

eTable 6. Perceived Health Benefits Across Viadin H Supplement Label Types

eAppendix. Surveys

This supplemental material has been provided by the authors to give readers additional information about their work.

© 2025 Assadourian JN et al. *JAMA Network Open*.

eFigure 1. Fish Oil "Supports Heart Health" Label

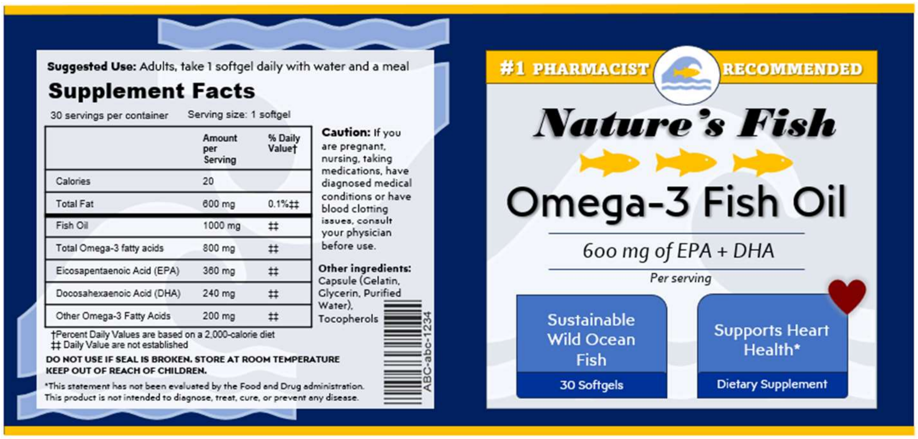

eFigure 2. Fish Oil "Supports Cognitive Function" Label

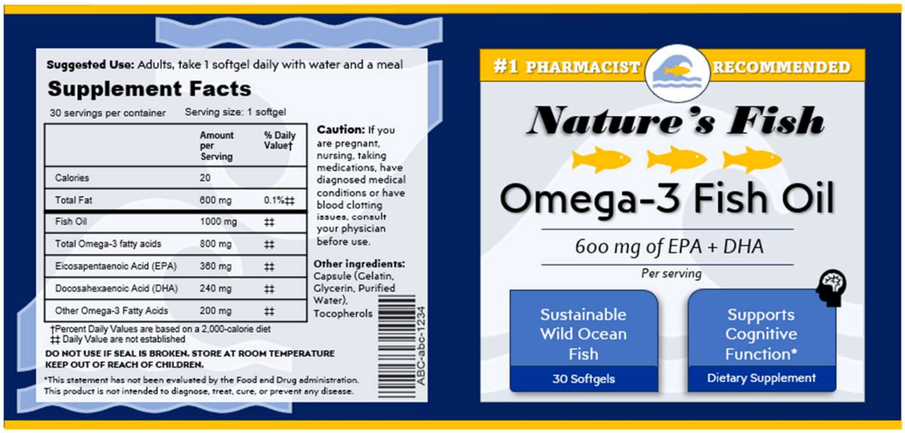

eFigure 3. Fish Oil Coronary Heart Disease Qualified Health Claim Label

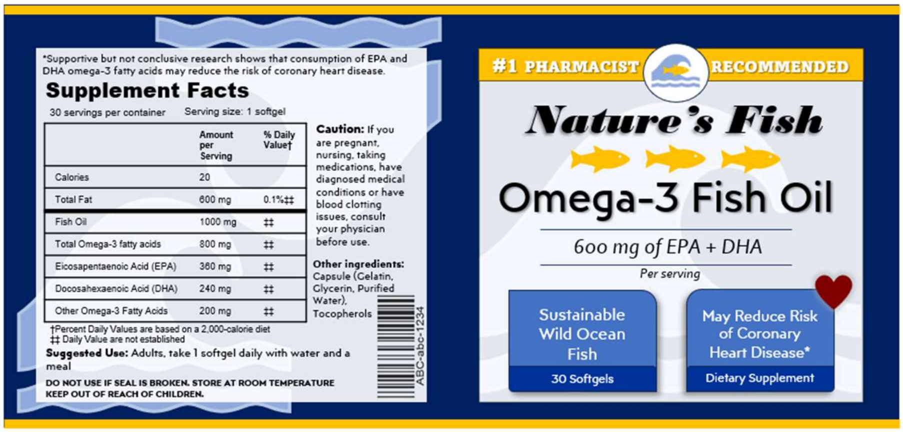

© 2025 Assadourian JN et al. *JAMA Network Open*.

eFigure 4. Fish Oil No Health Claim Label

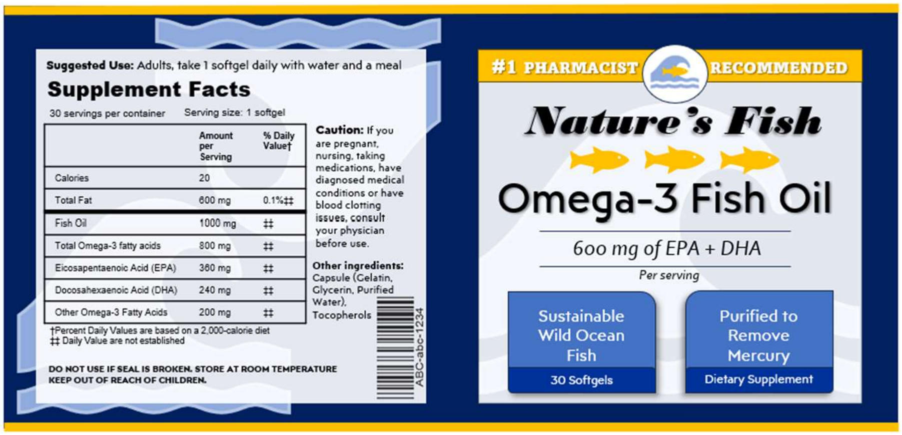

© 2025 Assadourian JN et al. *JAMA Network Open*.

eFigure 5. Viadin H Heart Health Label

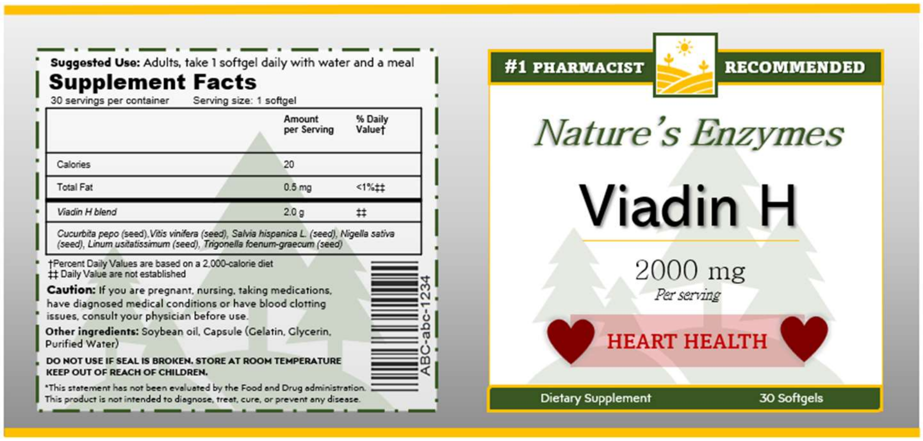

eFigure 6. Viadin H Supports Heart Function Label

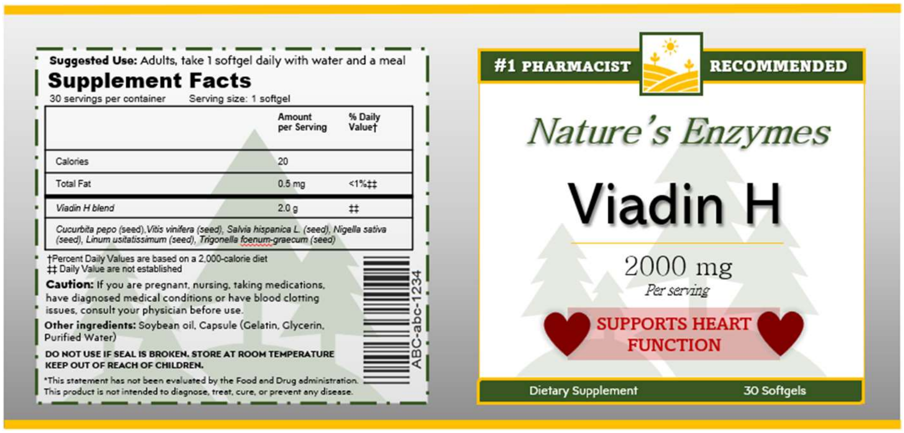

eFigure 7. Viadin H Brain Health Label

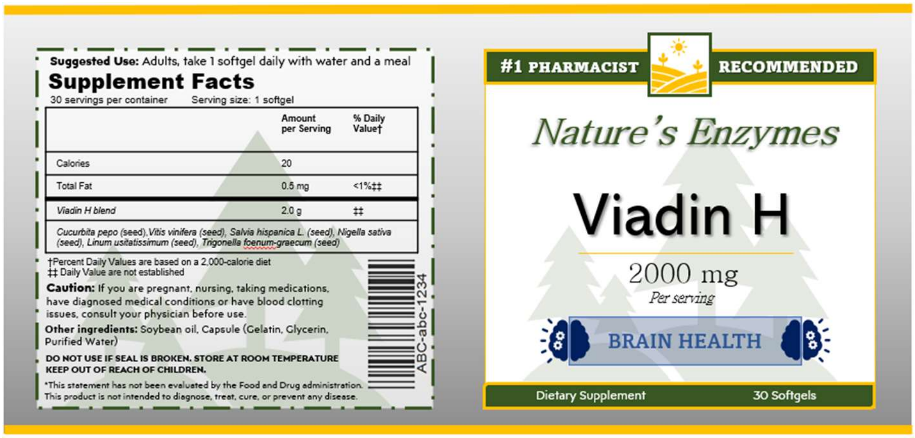

© 2025 Assadourian JN et al. *JAMA Network Open*.

eFigure 8. Viadin H Supports Cognitive Function Label

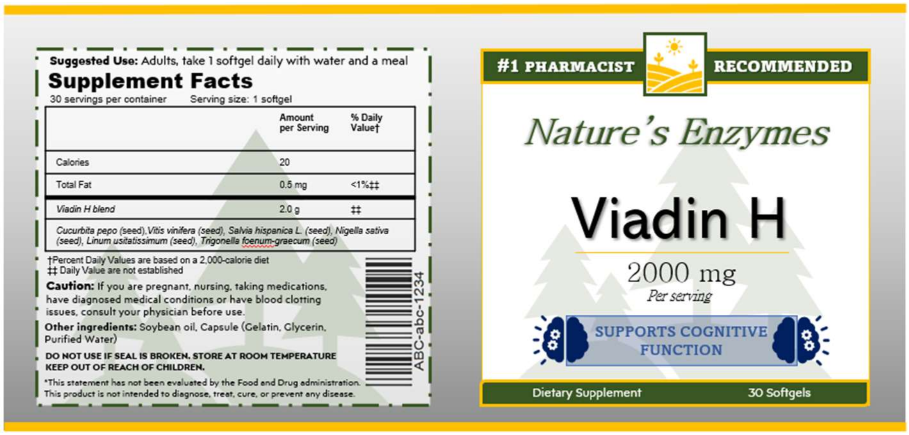

© 2025 Assadourian JN et al. *JAMA Network Open*.

eFigure 9. Percentage of Respondents That Responded That the Viadin H Supplement Would Treat or Prevent Specific Diseases by Randomized Label Statement Group

The figure shows the proportion of respondents randomized to either heart or neurologic related claims that reported that the supplement would "Likely" or "Very Likely" lead to the health outcome shown. Heart related labels included "Heart Health" or "Supports Heart Function." Cognition-related labels included "Brain Health" and "Supports Cognitive Function." p-values represent between-group difference based on Chi-Squared test.

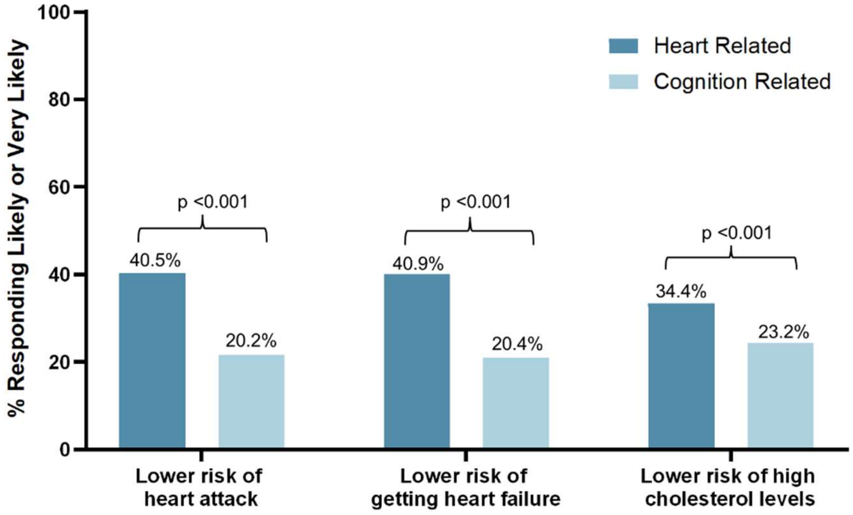

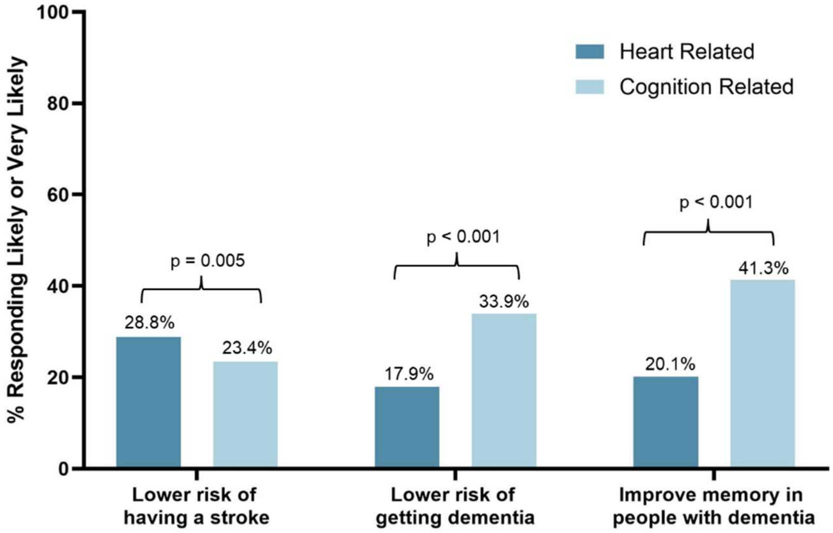

© 2025 Assadourian JN et al. *JAMA Network Open*.

eTable 1. Demographic Characteristics Across Different Fish Oil Label Claim Types

	"Supports Heart Health" Label (n=592)	Coronary Heart Disease QHC Label (n=541)	"Supports Cognitive Function" Label (n=538)	No Health Claim Label (n=568)
Age				
18-24	61 (10.5%)	57 (10.6%)	41 (7.8%)	45 (8.1%)
25-34	115 (19.8%)	92 (17.1%)	99 (18.8%)	114 (20.6%)
35-44	95 (16.4%)	112 (20.8%)	106 (20.1%)	94 (17.0%)
45-54	130 (22.4%)	114 (21.2%)	105 (19.9%)	124 (22.4%)
55-64	71 (12.2%)	77 (14.3%)	75 (14.2%)	80 (14.4%)
65-74	81 (13.9%)	65 (12.1%)	63 (12.0%)	69 (12.5%)
75 or older	28 (4.8%)	22 (4.1%)	38 (7.2%)	28 (5.1%)
Gender Identity				
Woman	307 (53.1%)	267 (50.6%)	279 (53.4%)	289 (53.3%)
Man	249 (43.1%)	240 (45.5%)	234 (44.7%)	232 (42.8%)
Transgender Woman	8 (1.4%)	2 (0.4%)	1 (0.2%)	1 (0.2%)
Transgender Man	1 (0.2%)	2 (0.4%)	3 (0.6%)	4 (0.7%)
Nonbinary	4 (0.7%)	3 (0.6%)	2 (0.4%)	3 (0.6%)
Other	2 (0.4%)	3 (0.6%)	1 (0.2%)	0 (0.0%)
Prefer Not to Answer	7 (1.2%)	11 (2.1%)	3 (0.6%)	13 (2.4%)
Race				
American Indian or Alaskan Native	9 (1.6%)	9 (1.7%)	8 (1.5%)	6 (1.1%)
Asian	65 (11.2%)	53 (10.0%)	61 (11.7%)	55 (10.1%)
Black or African American	41 (7.1%)	29 (5.5%)	29 (5.5%)	37 (6.8%)
Native Hawaiian or Pacific Islander	5 (0.9%)	5 (0.9%)	3 (0.6%)	2 (0.4%)
White	411 (71.0%)	393 (73.9%)	386 (73.8%)	394 (72.3%)
Multi-Racial	14 (2.4%)	15 (2.8%)	9 (1.7%)	12 (2.2%)
Other	13 (2.3%)	10 (1.9%)	11 (2.1%)	17 (3.1%)

© 2025 Assadourian JN et al. *JAMA Network Open*.

Prefer Not to Answer	21 (3.6%)	18 (3.4%)	16 (3.1%)	22 (4.0%)
Ethinicity				
Hispanic/Latinx	72 (12.9%)	80 (15.4%)	68 (13.3%)	83 (15.7%)
Education				
Some high school	13 (2.3%)	14 (2.6%)	12 (2.3%)	18 (3.3%)
High School/GED	78 (13.6%)	76 (14.3%)	73 (14.0%)	85 (15.7%)
Some College/AA	148 (25.7%)	109 (20.5%)	142 (27.3%)	129 (23.8%)
College Graduate	149 (25.9%)	184 (34.6%)	153 (29.4%)	146 (26.9%)
Graduate Degree	175 (30.4%)	142 (26.7%)	137 (26.3%)	152 (28.0%)
Prefer Not to Answer	12 (2.1%)	7 (1.3%)	4 (0.8%)	12 (2.2%)
Income				
Less than $20,000	65 (11.4%)	49 (9.3%)	58 (11.2%)	64 (11.8%)
$20,000 to $39,999	82 (14.3%)	86 (16.3%)	85 (16.4%)	78 (14.4%)
$40,000 to $59,999	92 (16.1%)	73 (13.8%)	92 (17.7%)	80 (14.7%)
$60,000 to $79,999	66 (11.5%)	72 (13.6%)	59 (11.4%)	77 (14.2%)
$80,000 to $99,999	46 (8.0%)	56 (10.6%)	52 (10.0%)	50 (9.2%)
$100,000 to $149,999	101 (17.7%)	70 (13.2%)	88 (17.0%)	96 (17.7%)
$150,000 or more	74 (12.9%)	80 (15.1%)	47 (9.1%)	58 (10.7%)
Prefer Not to Answer	46 (8.0%)	43 (8.1%)	38 (7.3%)	40 (7.4%)
Chronic Medical Conditions				
High cholesterol	169 (28.6%)	161 (29.8%)	162 (30.1%)	176 (31.0%)
Diabetes	99 (16.7%)	94 (17.4%)	112 (20.8%)	93 (16.4%)
Prior Heart Attack, Heart Artery stent, or Bypass Surgery	77 (13.0%)	66 (12.2%)	70 (13.0%)	83 (14.6%)

© 2025 Assadourian JN et al. *JAMA Network Open*.

Congestive heart failure	45 (7.6%)	43 (8.0%)	45 (8.4%)	52 (9.2%)
Supplement Use				
Any Supplement Use	509 (86.0%)	458 (84.7%)	469 (87.2%)	496 (87.3%)
Multivitamin	315 (53.2%)	284 (52.5%)	261 (48.5%)	314 (55.3%)
Vitamin D	315 (53.2%)	290 (53.6%)	307 (57.1%)	304 (53.5%)
Fish Oil	258 (43.6%)	238 (44.0%)	214 (39.8%)	231 (40.8%)
Calcium	149 (25.2%)	137 (25.3%)	139 (25.8%)	140 (24.7%)
Magnesium	144 (24.3%)	123 (22.7%)	127 (23.6%)	135 (23.8%)
Vitamin B12	133 (22.5%)	137 (25.3%)	132 (24.5%)	115 (20.3%)
Iron	96 (16.2%)	81 (15.0%)	77 (14.3%)	89 (15.7%)
B Complex	82 (13.9%)	79 (14.6%)	71 (13.2%)	61 (10.7%)
CoQ10	50 (8.5%)	48 (8.9%)	40 (7.4%)	43 (7.6%)
Other	45 (7.6%)	30 (5.6%)	33 (6.1%)	49 (8.6%)

© 2025 Assadourian JN et al. *JAMA Network Open*.

eTable 2. Demographic Characteristics Across Different Viadin H Label Claim Types

	"Heart Health" Label (n=525)	*"Supports Heart Function" Label (n=556)*	*"Brain Health" Label (n=563)*	*"Supports Cognitive Function" Label (n=536)*
Age				
18-24	46 (8.9%)	49 (8.9%)	46 (8.3%)	51 (9.7%)
25-34	73 (14.1%)	99 (18.0%)	73 (13.2%)	76 (14.4%)
35-44	102 (19.7%)	101 (18.4%)	134 (24.2%)	113 (21.4%)
45-54	147 (28.3%)	113 (20.6%)	128 (23.1%)	122 (23.1%)
55-64	68 (13.1%)	79 (14.4%)	78 (14.1%)	71 (13.5%)
65-74	59 (11.4%)	74 (13.5%)	69 (12.5%)	67 (12.7%)
75 or older	24 (4.6%)	35 (6.4%)	26 (4.7%)	28 (5.3%)
Gender Identity				
Woman	271 (53.1%)	277 (51.1%)	281 (51.4%)	256 (49.0%)
Man	231 (45.3%)	241 (44.5%)	247 (45.2%)	252 (48.3%)
Transgender Woman	2 (0.4%)	5 (0.9%)	3 (0.6%)	4 (0.8%)
Transgender Man	0 (0.0%)	3 (0.6%)	4 (0.7%)	1 (0.2%)
Nonbinary	1 (0.2%)	2 (0.4%)	4 (0.7%)	3 (0.6%)
Other	0 (0.0%)	3 (0.6%)	1 (0.2%)	0 (0.0%)
Prefer Not to Answer	5 (1.0%)	11 (2.0%)	7 (1.3%)	6 (1.2%)
Race				
American Indian or Alaskan Native	13 (2.5%)	10 (1.8%)	12 (2.2%)	5 (1.0%)
Asian	50 (9.7%)	57 (10.3%)	54 (9.8%)	48 (9.1%)
Black or African American	48 (9.3%)	44 (8.0%)	32 (5.8%)	41 (7.8%)
Native Hawaiian or Pacific Islander	3 (0.6%)	2 (0.4%)	7 (1.3%)	3 (0.6%)
White	363 (70.2%)	387 (70.1%)	403 (72.9%)	397 (75.5%)
Multi-Racial	8 (1.6%)	16 (2.9%)	11 (2.0%)	4 (0.8%)
Other	16 (3.1%)	19 (3.4%)	16 (2.9%)	13 (2.5%)

© 2025 Assadourian JN et al. *JAMA Network Open*.

Prefer Not to Answer	16 (3.1%)	17 (3.1%)	18 (3.3%)	15 (2.9%)
Ethinicity				
Hispanic/Latinx	75 (15.2%)	96 (18.2%)	71 (13.4%)	78 (15.7%)
Education				
Some high school	15 (2.9%)	22 (4.1%)	13 (2.4%)	15 (2.9%)
High School/GED	77 (15.0%)	74 (13.7%)	73 (13.4%)	78 (14.9%)
Some College/AA	117 (22.8%)	130 (24.0%)	147 (26.9%)	113 (21.7%)
College Graduate	142 (27.7%)	155 (28.7%)	160 (29.3%)	148 (28.4%)
Graduate Degree	154 (30.0%)	152 (28.1%)	149 (27.2%)	158 (30.3%)
Prefer Not to Answer	8 (1.6%)	8 (1.5%)	5 (0.9%)	10 (1.9%)
Income				
Less than $20,000	44 (8.6%)	51 (9.4%)	53 (9.7%)	63 (12.1%)
$20,000 to $39,999	58 (11.3%)	85 (15.6%)	79 (14.5%)	64 (12.2%)
$40,000 to $59,999	85 (16.6%)	78 (14.3%)	78 (14.3%)	70 (13.4%)
$60,000 to $79,999	74 (14.5%)	75 (13.8%)	86 (15.8%)	65 (12.4%)
$80,000 to $99,999	57 (11.1%)	49 (9.0%)	60 (11.0%)	55 (10.5%)
$100,000 to $149,999	95 (18.6%)	95 (17.4%)	84 (15.4%)	109 (20.8%)
$150,000 or more	63 (12.3%)	67 (12.3%)	65 (11.9%)	60 (11.5%)
Prefer Not to Answer	36 (7.0%)	45 (8.3%)	40 (7.3%)	37 (7.1%)
Supplement Use				
Any Supplement Use	417 (81.1%)	432 (80.0%)	438 (81.0%)	400 (77.1%)
Multivitamin	274 (52.2%)	289 (52.0%)	293 (52.0%)	259 (48.3%)
Vitamin D	259 (49.3%)	261 (46.9%)	296 (52.6%)	265 (49.4%)
Fish Oil	128 (24.4%)	133 (23.9%)	144 (25.6%)	140 (26.1%)
Vitamin C	136 (25.9%)	119 (21.4%)	123 (21.9%)	108 (20.2%)

© 2025 Assadourian JN et al. *JAMA Network Open*.

Calcium	114 (21.7%)	118 (21.2%)	111 (19.7%)	113 (21.1%)
Vitamin B12	114 (21.7%)	114 (20.5%)	113 (20.1%)	110 (20.5%)
Magnesium	102 (19.4%)	111 (20.0%)	113 (20.1%)	108 (20.2%)
Iron	70 (13.3%)	86 (15.5%)	71 (12.6%)	72 (13.4%)
B Complex	56 (10.7%)	62 (11.2%)	74 (13.1%)	64 (11.9%)
Turmeric	60 (11.4%)	61 (11.0%)	63 (11.2%)	65 (12.1%)
Zinc	61 (11.6%)	60 (10.8%)	71 (12.6%)	49 (9.1%)
Vitamin E	39 (7.4%)	52 (9.4%)	60 (10.7%)	54 (10.1%)
Collagen	49 (9.3%)	46 (8.3%)	56 (10.0%)	37 (6.9%)
Vitamin A	40 (7.6%)	45 (8.1%)	55 (9.8%)	44 (8.2%)
Elderberry	35 (6.7%)	44 (7.9%)	40 (7.1%)	25 (4.7%)
CoQ10	40 (7.6%)	28 (5.0%)	43 (7.6%)	30 (5.6%)
Other	18 (3.4%)	22 (4.0%)	32 (5.7%)	24 (4.5%)
Lutein Zeaxanthin	17 (3.2%)	19 (3.4%)	17 (3.0%)	14 (2.6%)

© 2025 Assadourian JN et al. *JAMA Network Open*.

eTable 3. Baseline Perceived Health Benefits of Fish Oil Among Fish Oil Users and Non–Fish Oil Users

Familiarity of Fish Oil				
	Overall (n=2275)	Fish Oil Users (n=941)	Non-Users (n=1296)	p-value
I have never heard of it	100 (4.4%)	57 (6.1%)	41 (3.2%)	<0.001
I have heard of it, but I do not know what it is	328 (14.4%)	87 (9.3%)	235 (18.1%)	
I have heard of it and know something about it	956 (42.0%)	189 (20.1%)	746 (57.6%)	
I am very familiar with it	867 (38.1%)	604 (64.2%)	256 (19.8%)	
Not sure/Prefer not to answer	24 (1.1%)	4 (0.4%)	18 (1.4%)	
Aware of Any Health Benefits?				
Yes	1,456 (78.8%)	747 (93.7%)	696 (68.2%)	<0.001
No	188 (10.2%)	26 (3.3%)	157 (15.4%)	
Not Sure	194 (10.5 %)	24 (3.0%)	167 (16.4%)	
Missing Data	9 (0.4%)	---	---	

© 2025 Assadourian JN et al. *JAMA Network Open*.

eTable 4. Perceived Health Benefits Across Fish Oil Label Types

	"Supports Heart Health" Label (n=592)	p-value	*Coronary Heart Disease QHC Label (n=541)*	p-value	*"Supports Cognitive Function" Label (n=538)*	p-value	*No Health Claim Label (n=568)*
Lower risk of heart attack	370 (62.5%)	0.003	326 (60.3%)	0.032	255 (47.4%)	0.031	306 (53.9%)
Lower risk of getting heart failure	349 (59.0%)	0.005	326 (60.3%)	0.001	248 (46.1%)	0.125	288 (50.7%)
Lower risk of high cholesterol levels	375 (63.3%)	0.960	338 (62.5%)	0.802	313 (58.2%)	0.087	359 (63.2%)
Lower risk of having a stroke	319 (53.9%)	0.012	269 (49.7%)	0.280	230 (42.8%)	0.213	264 (46.5%)
Lower risk of getting dementia	212 (35.8%)	0.182	193 (35.7%)	0.176	255 (47.4%)	0.009	225 (39.6%)
Improve memory in people with dementia	229 (38.7%)	0.528	205 (37.9%)	0.375	258 (48.0%)	0.012	230 (40.5%)
Lower risk of osteoporosis/low bone density	157 (26.5%)	0.274	140 (25.9%)	0.190	150 (27.9%)	0.576	167 (29.4%)
Lower risk of getting cancer	216 (36.5%)	0.247	209 (38.6%)	0.693	211 (39.2%)	0.846	226 (39.8%)

© 2025 Assadourian JN et al. *JAMA Network Open*.

eTable 5. Perceived Health Benefits Between Fish Oil Users vs Nonusers Across Fish Oil Label Types

Fish Oil Supplement Users							
	"Supports Heart Health" Label (n=258)	*p-value*	*Coronary Heart Disease QHC Label (n=238)*	*p-value*	*"Supports Cognitive Function" Label (n=214)*	*p-value*	*No Health Claim Label (n=231)*
Lower risk of heart attack	183 (70.9%)	0.766	171 (71.9%)	0.608	151 (70.6%)	0.842	161 (69.7%)
Lower risk of getting heart failure	168 (65.1%)	0.442	174 (73.1%)	0.262	146 (68.2%)	0.969	158 (68.4%)
Lower risk of high cholesterol levels	199 (77.1%)	0.925	182 (76.5%)	0.793	163 (76.2%)	0.741	179 (77.5%)
Lower risk of having a stroke	178 (69.0%)	0.249	154 (64.7%)	0.886	137 (64.0%)	0.991	148 (64.1%)
Lower risk of getting dementia	137 (53.1%)	0.115	130 (54.6%)	0.224	139 (65.0%)	0.298	139 (60.2%)
Improve memory in people with dementia	143 (55.4%)	0.442	131 (55.0%)	0.402	134 (62.6%)	0.419	136 (58.9%)
Lower risk of osteoporosis/low bone density	137 (53.1%)	0.199	133 (55.9%)	0.512	121 (56.5%)	0.619	136 (58.9%)
Lower risk of getting cancer	109 (42.3%)	0.115	101 (42.4%)	0.133	103 (48.1%)	0.797	114 (49.4%)
Non-Fish Oil Supplements Users							
	"Supports Heart Health" Label (n=334)	p-value	*Coronary Heart Disease QHC Label (n=303)*	p-value	*"Supports Cognitive Function" Label (n=324)*	p-value	*No Health Claim* Label (n=335)

© 2025 Assadourian JN et al. *JAMA Network Open*.

Lower risk of heart attack	187 (56.0%)	0.001	155 (51.2%)	0.047	104 (32.1%)	0.003	145 (43.3%)
Lower risk of getting heart failure	181 (54.2%)	<0.001	152 (50.2%)	0.004	102 (31.5%)	0.049	130 (38.8%)
Lower risk of high cholesterol levels	176 (52.7%)	0.788	156 (51.5%)	0.570	150 (46.3%)	0.056	180 (53.7%)
Lower risk of having a stroke	141 (42.2%)	0.044	115 (38.0%)	0.383	93 (28.7%)	0.102	116 (34.6%)
Lower risk of getting dementia	75 (22.5%)	0.331	63 (20.8%)	0.146	116 (35.8%)	0.005	86 (25.7%)
Improve memory in people with dementia	86 (25.8%)	0.500	74 (24.4%)	0.298	124 (38.3%)	0.005	94 (28.1%)
Lower risk of osteoporosis/low bone density	79 (23.7%)	0.339	76 (25.1%)	0.608	90 (27.8%)	0.793	90 (26.9%)
Lower risk of getting cancer	48 (14.4%)	0.676	39 (12.9%)	0.339	47 (14.5%)	0.715	52 (15.5%)

© 2025 Assadourian JN et al. *JAMA Network Open*.

eTable 6. Perceived Health Benefits Across Viadin H Supplement Label Types

	"Heart Health" Label 24.1% (n=525)	*"Supports Heart Function" Label 25.5% (n=556)*	*"Brain Health" Label 25.8% (n=556)*	*"Supports Cognitive Function" Label 24.6% (n=536)*	*p-value*
Lower risk of heart attack	209 (40.0%)	225 (40.5%)	113 (20.2%)	124 (23.3%)	<0.001
Lower risk of getting heart failure	205 (39.2%)	227 (40.9%)	114 (20.4%)	115 (21.6%)	<0.001
Lower risk of high cholesterol levels	169 (32.3%)	191 (34.4%)	130 (23.2%)	136 (25.6%)	<0.001
Lower risk of having a stroke	152 (29.1%)	158 (28.5%)	139 (24.8%)	117 (22.0%)	0.028
Lower risk of getting dementia	98 (18.7%)	95 (17.1%)	193 (34.5%)	177 (33.3%)	<0.001
Improve memory in people with dementia	105 (20.1%)	112 (20.2%)	219 (39.1%)	232 (43.6%)	<0.001
Lower risk of osteoporosis/ low bone density	95 (18.2%)	106 (19.1%)	99 (17.7%)	99 (18.6%)	0.938
Lower risk of getting cancer	99 (18.9%)	113 (20.4%)	126 (22.5%)	123 (23.1%)	0.310

© 2025 Assadourian JN et al. *JAMA Network Open*.

eAppendix. Surveys

Fish Oil Survey Questions

Welcome

Thank you very much for participating in our survey. Your response is voluntary and will remain anonymous.

Please answer the questions to the best of your ability. If you do not know an answer, please select the answer choice “Not sure /Prefer Not to Answer.”

Page Break

Based on your understanding, please answer the following questions:

Q) How much do you know about fish oil supplements?

a) I've never heard of it
b) I've heard of it, but I do not know what it is
c) I have heard of it and know something about what it is
d) I am very familiar with it
e) Not sure/prefer not to answer

Q) Are you aware of any health benefits of fish oil supplements?

a) Yes
b) No

Page Break

Please take a moment to look at this label. Note that the label information you see may or may not be the same as what you would see in the market.

Insert label image here

Q) On a scale of 1 to 5, how likely is that the product may help lower or reduce the risk of any of these health problems.

Scale: 1. Not at all likely, 2. Unlikely, 3. Neither likely nor unlikely, 4. Likely, 5. Very Likely, Not sure/Prefer not to answer

- Getting heart failure
- Getting dementia
- Having osteoporosis/low bone density
- Getting cancer

© 2025 Assadourian JN et al. *JAMA Network Open*.

- Having a heart attack
- Having a stroke

Q) On a scale of 1 to 5, how likely is that the product may help lower or reduce the risk of any of these health problems.

Scale: 1. Not at all likely, 2. Unlikely, 3. Neither likely nor unlikely, 4. Likely, 5. Very Likely, Not sure/Prefer not to answer

- Lower high cholesterol levels
- Improve memory in people with dementia

Q) Do you regularly take any fish oil supplements?

- Yes
- No

Q) Do you regularly take any other dietary supplement(s) or vitamins?

- Yes
- No
- Prefer not to Answer

If so, mark all that apply?

- Multivitamin
- Vitamin D
- Fish Oil or omega 3
- Calcium
- Magnesium
- Iron
- Vitamin B12
- B Complex
- Co-Q-10
- None of the above
- Other (please specify)

The following questions are optional

Q) Please indicate if you have any of the following chronic medical conditions:

- Diabetes
- Prior heart attack, heart artery stent, or bypass surgery

© 2025 Assadourian JN et al. *JAMA Network Open*.

- Congestive heart failure
- High cholesterol

Q) What is your age?

- 18-24
- 25-34
- 35-44
- 45-54
- 55-64
- 65-75
- 75 or older

Q) Indicate your race. Mark all that apply.

- White/Caucasian
- Black or African American
- Asian/Pacific Islander
- American Indian or Alaskan Native
- Multi-racial
- Other
- Prefer not to answer

Q) Are you Hispanic/Latinx?

- Yes
- No
- Prefer not to answer

Q) Indicate your gender identity.

- Female
- Non-binary
- Male
- Other
- Prefer not to answer

Q) What is the highest level of education you attained?

- Some high school
- High school/GED or equivalent
- Some college or associate degree

© 2025 Assadourian JN et al. *JAMA Network Open*.

- College graduate
- Graduate degree
- Prefer not to answer

Q) What is your annual income?

- Less than $20,000
- $20,000 to $39,999
- $40,000 to $59,999
- $60,000 to $79,999
- $80,000 to $99,999
- $100,000 to $149,999
- $150,000 or more
- Prefer not to answer

© 2025 Assadourian JN et al. *JAMA Network Open*.

Viadin H Survey Questions

Welcome

Thank you very much for participating in our survey. Your response is voluntary and will remain anonymous.

Please answer the questions to the best of your ability. If you do not know an answer, please select the answer choice "Not sure /Prefer Not to Answer."

Page Break

Please take a moment to look at this label. Note that the label information you see may or may not be the same as what you would see in the market.

Insert label image here

Q) On a scale of 1 to 5, how likely is that the product may help lower or reduce the risk of any of these health problems.

Scale: 1. Not at all likely, 2. Unlikely, 3. Neither likely nor unlikely, 4. Likely, 5. Very Likely, Not sure/Prefer not to answer

- Getting heart failure
- Getting dementia
- Having osteoporosis/low bone density
- Getting cancer
- Having a heart attack
- Having a stroke

Q) On a scale of 1 to 5, how likely is that the product may help lower or reduce the risk of any of these health problems.

Scale: 1. Not at all likely, 2. Unlikely, 3. Neither likely nor unlikely, 4. Likely, 5. Very Likely, Not sure/Prefer not to answer

- Lower high cholesterol levels
- Improve memory in people with dementia

Q) Do you regularly take any other dietary supplement(s) or vitamins? If so, mark all that apply?

- Multivitamin
- Vitamin D
- Fish Oil or omega 3

© 2025 Assadourian JN et al. *JAMA Network Open*.

- Calcium
- Magnesium
- Iron
- Vitamin B12
- B Complex
- Co-Q-10
- Vitamin C
- Turmeric
- Zinc
- Vitamin E
- Collagen
- Vitamin A
- Elderberry
- Lutein Zeaxanthin
- None of the above
- Other (please specify)

The following questions are optional

Q) What is your age?

- 18-24
- 25-34
- 35-44
- 45-54
- 55-64
- 65-75
- 75 or older

Q) Indicate your race. Mark all that apply.

- White/Caucasian
- Black or African American
- Asian/Pacific Islander
- American Indian or Alaskan Native
- Multi-racial
- Other
- Prefer not to answer

Q) Are you Hispanic/Latinx?

© 2025 Assadourian JN et al. *JAMA Network Open*.

- Yes
- No
- Prefer not to answer

Q) Indicate your gender identity.

- Female
- Non-binary
- Male
- Other
- Prefer not to answer

Q) What is the highest level of education you attained?

- Some high school
- High school/GED or equivalent
- Some college or associate degree
- College graduate
- Graduate degree
- Prefer not to answer

Q) What is your annual income?

- Less than $20,000
- $20,000 to $39,999
- $40,000 to $59,999
- $60,000 to $79,999
- $80,000 to $99,999
- $100,000 to $149,999
- $150,000 or more
- Prefer not to answer

© 2025 Assadourian JN et al. *JAMA Network Open*.
