# Supplementary material for: Label Statements and Perceived Health Benefits of Dietary Supplements
Source: JAMA Netw Open. 2025 Sep 22;8(9):e2533118. doi: 10.1001/jamanetworkopen.2025.33118 (PMC12455376; doi:10.1001/jamanetworkopen.2025.33118)
Supplement: Supplement 2. — Data Sharing Statement [file jamanetwopen-e2533118-s002.pdf]

Data Sharing Statement

Assadourian. Label Statements and Perceived Health Benefits of Dietary Supplements. *JAMA Netw Open*. Published September 22, 2025. doi:10.1001/jamanetworkopen.2025.33118

Data
Data available: Yes
Data types: Deidentified participant data
How to access data: Deidentified participant data can be made available upon reasonable request to researchers with appropriate institutional data sharing agreements in place.
When available: With publication

Supporting Documents
Document types: None

Additional Information
Who can access the data: Deidentified participant data can be made available upon reasonable request to researchers with appropriate institutional data sharing agreements in place.
Types of analyses: For IRB approved research
Mechanisms of data availability: With investigator support after approval and with signed data access agreement.
